# DCCAM-MRNet: Mixed Residual Connection Network with Dilated Convolution and Coordinate Attention Mechanism for Tomato Disease Identification

**DOI:** 10.1155/2022/4848425

**Published:** 2022-04-15

**Authors:** Yujian Liu, Yaowen Hu, Weiwei Cai, Guoxiong Zhou, Jialei Zhan, Liujun Li

**Affiliations:** ^1^College of Computer & Information Engineering, Central South University of Forestry and Technology, Changsha 410004, China; ^2^School of Artificial Intelligence and Computer Science, Jiangnan University, Wuxi 214122, China; ^3^Graduate School, Northern Arizona University, Flagstaff, AZ 86011, USA; ^4^Missouri University of Science & Technology, Department of Civil, Architectural and Environmental Engineering, Rolla, MO 65409, USA

## Abstract

Tomato is an important and fragile crop. During the course of its development, it is frequently contaminated with bacteria or viruses. Tomato leaf diseases may be detected quickly and accurately, resulting in increased productivity and quality. Because of the intricate development environment of tomatoes and their inconspicuous disease spot features and small spot area, present machine vision approaches fail to reliably recognize tomato leaves. As a result, this research proposes a novel paradigm for detecting tomato leaf disease. The INLM (integration nonlocal means) filtering algorithm, for example, decreases the interference of surrounding noise on the features. Then, utilizing ResNeXt50 as the backbone, we create DCCAM-MRNet, a novel tomato image recognition network. Dilated Convolution (DC) was employed in STAGE 1 of the DCCAM-MRNet to extend the network's perceptual area and locate the scattered disease spots on tomato leaves. The coordinate attention (CA) mechanism is then introduced to record cross-channel information and direction- and position-sensitive data, allowing the network to more accurately detect localized tomato disease spots. Finally, we offer a mixed residual connection (MRC) technique that combines residual block (RS-Block) and transformed residual block (TR-Block) (TRS-Block). This strategy can increase the network's accuracy while also reducing its size. The DCCAM-classification MRNet's accuracy is 94.3 percent, which is higher than the existing network, and the number of parameters is 0.11 M lesser than the backbone network ResNeXt50, according to the experimental results. As a result, combining INLM and DCCAM-MRNet to identify tomato diseases is a successful strategy.

## 1. Introduction

Tomatoes are a globally important vegetable crop [1]. However, diseases can harm tomatoes, reducing their quality and yield. Leaf mold, Septoria leaf spot, yellow leaf curl virus, tomato mosaic virus, target spot, and two-spotted spider mite are all common diseases of tomato foliage. In their early stages, these diseases produce small irregular-shaped spots that are dispersed and difficult to identify. In comparison, the late stage of the disease, with its distinctive spots and large spot areas, is easier to identify. Still, it is discovered late, resulting in a significant loss of tomato quality and yield. As a result, disease detection technology for tomatoes is critical. However, traditional manual identification and knowledge base-based expert system methods are highly subjective and reliant on farmers and experts [2]. Lesions on tomato leaves vary in shape and are insufficiently characterized. Although certain diseases have distinctive spots in terms of shape and color, they are difficult to distinguish with the naked eye because of the small spot area and require magnifying equipment, such as magnifying glasses or microscopes for observation. Thus, detecting tomato leaf diseases quickly and accurately and implementing appropriate control measures are critical to ensuring tomato production.

Disease-based leaf recognition methods are a popular research direction in computer vision and image processing [3–5]. Numerous studies have successfully combined image processing and traditional machine learning techniques, resulting in significant application value [6, 7]. However, most disease recognition algorithms extract image features via multiple filters [8]. The extraction process is tedious and frequently selects for recognition objects with noticeable disease features and concentrated disease areas. As a result, this traditional recognition method cannot extract disease features from tomatoes. Along with the explosion in data volume and the advancement of computer hardware, deep learning has made significant strides in image recognition [9, 10]. Many researchers prefer convolutional neural networks because of their three primary features: local perception, multiple convolutional kernels, and parameter sharing. For instance, Bedi and Gole [11] proposed a novel hybrid model based on a convolutional autoencoder (CAE) network and convolutional neural network (CNN) for automatic plant disease detection. Reference [12] used a convolutional neural network to extract features from a large dataset containing 14,828 images of tomato leaves infected with nine diseases. They visualized the results, achieving a 99.18 percent accuracy rate. Abbas et al. [13] used GAN and transfer learning to identify and classify tomato plant disease, achieving an average classification accuracy of 99.35 percent. Su et al. [14] separately fed one-dimensional spectral and three-dimensional hyperspectral images of ripe strawberries into a ResNet classification network. Both inputs were more than 84 percent accurate in the ResNet classification network. However, the error persisted even with the prepared dataset, as the shooting environment and equipment constrained the image quality. To ensure successful recognition, the features of spots on tomato leaves are compared to those of typical pests and diseases. It frequently falls victim to locally optimal solutions and gradient disappearance, resulting in low recognition accuracy. As a result, the main problems of the study are as follows: (1) the images in the tomato leaf disease dataset are gathered from a variety of sources, including the internet and the demonstration base of Hunan Vegetable Research Institute, and they suffer from a complex background and uneven quality. When the network is fed the original images, it can extract features from the training set. Nonetheless, the network may extract blurred features from the original images (e.g., speckled objects, such as dust and dirt) as features, resulting in incorrect extraction. (2) Common tomato leaf diseases produce subtle differences in the appearance of leaves, such as spots and slight yellowing, complicating disease recognition. Additionally, the disease areas in the disease images are small and scattered, complicating feature extraction. (3) In cases where some disease features are not readily apparent, more subtle features must be extracted. Increasing the number of network layers in the model can enhance the recognition ability of the network. Nonetheless, the resulting issue is that the network will be more difficult to train. Given that the subject of this study is tomato leaf disease, a prolonged training period will quickly result in additional disease damage to tomatoes. As a result, the network requires high accuracy, few parameters, and rapid model convergence.

Buades et al. [15] proposed the nonlocal mean filtering algorithm to address the issue of tomato leaf disease images being susceptible to background interference and blurred features. Because of its novel comparison of local similarity, it outperforms other traditional algorithms in terms of filtering effect and better preserves image edge details. Recent years have seen a surge in improvements based on this classic filtering algorithm. Dore and Cheriet [16] enhanced the denoising effect of the NL-Means algorithm by incorporating a robust regression with fixed smoothing parameters. It significantly reduces the blurring caused by weight. To address the issue of similarity accuracy degradation of this algorithm in the presence of harsh noise, Guo et al. [17] incorporated the feature similarity of the multichannel filter into the NL-means filter. The experimental results indicate that this filtering method outperforms the more traditional NL-means and wavelet-based filtering methods in terms of filtering effect. On the other hand, Kanoun et al. [18] proposed the KS-NLM filtering algorithm, which combines the NL-Means filter with anisotropic weighting to handle the central pixels of the patch better. The filtering algorithms above based on NL-Means perform better than NL-mean at denoising. Nonetheless, it does not satisfactorily address the high computational complexity and lengthy procedure of the NL-mean algorithm. The INLM filtering algorithm performs admirably well in terms of filtering. Its computational complexity is significantly lower than NL-Means, and its convergence speed is considerably faster, resulting in a shorter filtering time.

Das et al. [19] used a more complex network architecture to boost classification accuracy to 95.91%, resolving the issue of neural networks having difficulty identifying features associated with heart diseases. Brahimi et al. [12] demonstrated that increasing the number of layers in neural networks improves model performance, and nine tomato disease regions were identified as a result. However, increasing the number of network layers allows for more accurate features extraction. Nonetheless, there are two disadvantages: (1) when the number of layers in the neural network exceeds a certain threshold, gradient explosion and disappearance occur. These factors jeopardize crop disease identification. (2) To extract more detailed features, a deep neural network must be designed. However, the number of parameters to compute increases when training a deep neural network, resulting in slow convergence. Based on the aforementioned issues, this paper proposes DCCAM-MRNet, which utilizes ResNeXt50 as the backbone network and its unique residual mechanism to avoid gradient explosion and disappearance problems. In STAGE 1 of the network, dilated convolution is introduced, as well as a coordinate attention mechanism is inserted between each 3 × 3 and 1 × 1 convolution to improve feature extraction for subtle diseases.

To address the difficulty of training deep models, Luo et al. [20] incorporated the highway network into a bidirectional gated recurrent unit. The attention mechanism is additionally utilized in an effort to assign the weights of key issues in the network structure. Peng et al. [21] proposed ResNet, which uses residual shortcut connection to combine the output of residual components with the input and uses the residual learning mechanism to solve the trainability problem of deep neural networks. Lu et al. [22] proposed a multistep linear structure based on the numerical solution of differential equations in 2017. They built it to examine a more efficient deep neural network called LM-ResNet, based on ResNet. The preceding three examples show how to find appropriate residual shortcut connections to guide the structural design of deep neural networks and how to set the appropriate initialization conditions for network weights and training parameters, which can help solve network trainability and model efficiency problems. The mixed residual connection approach proposed in this study is based on the Adams method of numerical solution of differential equations and comes in two flavors: RS-Block and TRS-Block. The DCCAM-MRNet network is built in such a way that it can continue to update the weights, achieve high learning accuracy, and make the network effective even when the magnitude of the gradient value update is minimal during the later phases of network training, thanks to ResNeXt's residual mechanism.

**Figure 1:**
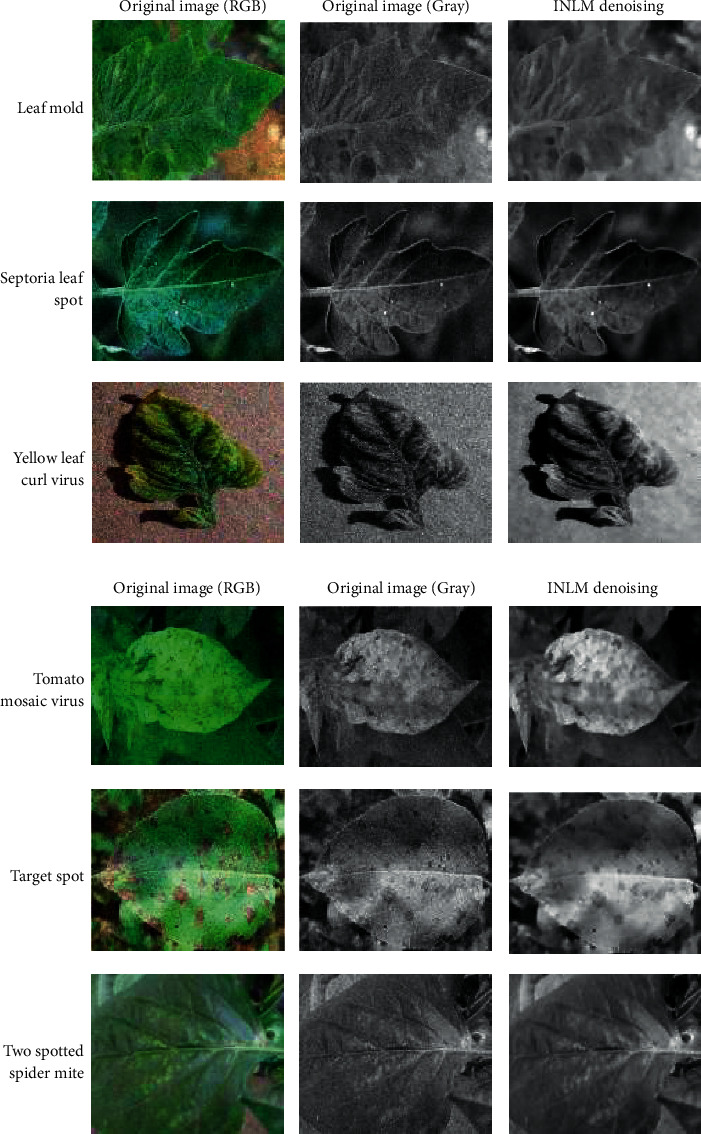
Comparison of original images and filtered images.

The contributions of this paper are as follows:

1. To reduce the impact of complex tomato planting background and fuzzy features of tomato leaf diseases on recognition accuracy, the INLM filtering algorithm is proposed in this paper. The INLM filtering algorithm reduces computational complexity after integrating the images, and it effectively overcomes the disadvantage of slow NL-Means computation. As shown in Figure 1, the quality of the images processed by the INLM filtering algorithm is improved compared with the original images. As shown in Table 1, the INLM filtering algorithm is 10 times better than the NL-means algorithm in filtering speed.
2. To extract the scattered and narrow disease spot features of tomato, the DCCAM-MRNet is proposed in this paper. (a) In STAGE 1 of the network, dilated convolution is used to identify the scattered diseases of tomato leaf to capture multiscale contextual information without changing the number of parameters. As shown in Table 2, the use of dilated convolution improves the ability to extract the feature and detection accuracy by 1.7% to RexNeXt50. (b) A coordinate attention mechanism, cochannel correlation, and remote dependence are introduced between 3 × 3 and 1 × 1 convolution for modeling, which enhanced the extraction of tomato microdisease features and increased recognition accuracy by 3.6% to RexNeXt50 (as shown in Table 3). (c) The residual block (RS-Block) and transformation block (TRS-Block) of the mixed residual connection method are used in this paper to improve the trainability of the DCCAM-MRNet structure. The similarity is that adjacent residual blocks are weighted and added to the current residual block, resulting in a more accurate extraction of features between adjacent layers. The distinction is that the TRS-Block utilizes the channel conversion function to match the input channel to the input channel. As shown in Tables 4 and 5, The recognition accuracy of DCCAM-MRNet is increased by 0.9% to RexNeXt50, and 0.11 M reduces its parameter count compared to the backbone network of RexNeXt50
3. Compared to conventional deep neural networks, the DCCAM-MRNet proposed in this paper accurately recognizes tomato leaf diseases (as shown in Table 6). Additionally, this network has fewer parameters than the backbone network ResNeXt50 and is more trainable.

**Figure 2:**
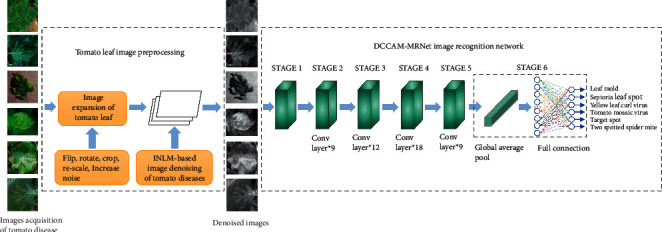
Principles of tomato disease identification.

As a result, this paper proposes a method for identifying tomato diseases that combines the INLM filtering algorithm and the DCCAM-MRNet. The identification principle is depicted in Figure 2. Firstly, the expanded dataset is passed through the INLM filtering algorithm, which reduces the influence of complex background and blurred features on the image, laying the groundwork for recognition and classification using the model. The processed dataset is then used to train and test the DCCAM-MRNet. To improve the ability of the model to extract features from the image set, the DCCAM-MRNet is enhanced with dilation convolution and coordinate attention mechanisms. The mixed residual connection method is used to increase the trainability of the network.

## 2. Materials and Methods

### 2.1. Data Acquisition

Datasets have been critical components of tomato leaf disease identification methods. The tomato leaf disease dataset in the Hunan Academy of Agricultural Sciences demonstration base was compiled using data from tomato greenhouse and the internet. We used a Nikon camera with a resolution of 4460 × 3740 in the tomato greenhouse. Leaf mold, Septoria leaf spot, yellow leaf curl virus, tomato mosaic virus, target spot, and two-spotted spider mite were included in the dataset. As illustrated in Table 7, these diseases cause irregular, colorful, scattered spots with indistinct margins, and disease features differ significantly between the early and late stages.

To alleviate the strain on the computer system caused by these images, each image in the tomato leaf disease dataset was compressed at resolution using the Matlab 2020b software. The compression specification for these images was 224 × 224, and they were imported into the computer in the jpg format. To avoid model overfitting and poor generalization performance because of its small number of training samples, we expanded the dataset using MATLAB by flipping, cropping, scaling, highlighting the images, and saving them in jpg format.

The expanded tomato leaf disease dataset contains 10,923 images of tomato leaf disease. This experiment separated the dataset into the training set of 7646 tomato leaf disease images, the validation set of 2185 tomato leaf disease images, and the test set of 1092 tomato leaf disease images in a 7 : 2 : 1 ratio to train and test the network.  Table 8 displays the comparison of the accuracy before and after pre-processing.  Table 9 shows the distribution of the six disease images in the dataset.

### 2.2. INLM Image Filtering

As shown in Table 1, the images of tomatoes taken in greenhouses have complex growing environments, while diseases such as leaf mold, yellow leaf curl virus, and target spot exhibit blurred features. Both issues will inevitably introduce noise into the collected images, degrading their quality. As a result, it is worthwhile to investigate measures to eliminate noise while retaining the images' essential features. The INLM algorithm fully exploits redundant information in the images, significantly preserves the details and textures of the original images during filtering, and accelerates the filtering process by integrating the images, thereby reducing the complexity of operations and filtering time. Because of its efficiency and simplicity, the INLM algorithm is used in this study.

#### 2.2.1. NL-Means Filtering Algorithm

The core idea of the NL-Means algorithm is to take a rectangular window of each pixel point domain and calculate the weighted sum of the pixel values of all the pixel points within the window, with the weights obeying a Gaussian distribution. It is similar to Gaussian filtering, but unlike Gaussian filtering [23], NL-Means use the similarity between the domain block of the current filtered point and the domain blocks of other points in the rectangular window to calculate the weights, and the greater the similarity, the greater the weights.

For an image, suppose *v*(*x*)=*u*(*x*)+*n*(*x*), where *v*(*x*) is the observed image with noise, *u*(*x*) is the real image without noise, and *n*(*x*) is the noise perturbation of pixel *x*. A noisy image *v*={*v*(*x*)*|x* ∈ *I*} is given, and *x* represents the position of the pixel in the image, *I* represents the set of individual pixels in the image, and *v*(*x*) represents the value corresponding to the position of the pixel *x*. The specific formula of the NL-Means filtering algorithm is as follows:

(1) $$\begin{array}{c} NL\left[v\left(x\right)\right]=\sum_{j\in I}w\left(x,y\right)v\left(y\right), \end{array}$$

*NL*[*v*(*x*)] represents the image filtered by the NL-Means filtering algorithm. *w*(*x*, *y*) is the similarity between pixel *x* and pixel *y*, and its value is the Gaussian kernel of the pixel value between each point in a domain. The specific formula is as follows:

(2) $$\begin{array}{c} w\left(x,y\right)=\frac{1}{z\left(x\right)}e^{-\left({\left\|v\left(N_{x}\right)-v\left(N_{y}\right)\right\|}_{2,a}^{2}/h^{2}\right)}. \end{array}$$

Among them,

(3) $$\begin{array}{c} Z\left(x\right)=\sum_{y}e^{-\left({\left\|v\left(N_{x}\right)-v\left(N_{y}\right)\right\|}_{2,a}^{2}/h^{2}\right)}, \end{array}$$

‖*v*(*N*_*x*_) − *v*(*N*_*y*_)‖_2,*a*_^2^ represents the Gaussian kernel. *v*(*N*_*x*_) refers to a domain in the image centered on *x*. *h* is the attenuation factor. The smaller the value of *h*, the lesser the influence of the weighted point on the current point, and the edge is maintained well, however, the noise is serious. On the contrary, the edge is maintained poorly, however, the image is smoother, and the filtering level is high.

#### 2.2.2. Tomato Disease Image Filtering Algorithm Based on INLM Algorithm

The size of the search area must be defined in the NL-Means algorithm, and the larger the search area, the greater the possibility of discovering similar pixels, however, the quantity of computation also increases exponentially. Assume the image is *N* × *N* pixels in size. With a color channel number of 3, a neighborhood window size of *k* × *k*, and a search box size of *n* × *n*, the complexity of the algorithm is o(3*N*^2^*k*^2^*n*^2^). Even in the original paper, the author defined the search area as a whole image, resulting in a few minutes of waste during the process of an image of 512 × 512 in size.

Based on the above description, it can be seen that the calculation complexity of the NL-Means algorithm is too high, and the program is very time-consuming, which is not conducive to practical application. After analyzing the formula, it was found that changing the similarity calculation between domains can reduce the time consumed.

If we first build an integral image for pixel differences, the equation is as follows:

(4) $$\begin{array}{c} S_{t}\left(x\right)=\sum_{\begin{array}{c} Z_{1}\leq x_{1}\\ Z_{2}\leq x_{2} \end{array}}S_{t}\left(Z\right),x\left(x_{1},x_{2}\right),\;S_{t}\left(x\right)={\left\|u\left(x\right)-u\left(x+t\right)\right\|}^{2}. \end{array}$$

Using this method to calculate the distance between the two domains *v*(*N*_*x*_) and *v*(*N*_*y*_) takes only a small amount of time, and the calculation equation is as follows:

(5) $$\begin{array}{c} {\left\|V\left(x\right)-V\left(y\right)\right\|}^{2}=\frac{1}{d^{2}}\left(S_{t}\left(x_{1}+ds\;,x_{2}+ds\right)+S_{t}\left(x_{1}-ds\right.\right.\\ -1\left.\left.x_{2}-ds-1\right)-S_{t}\left(x_{1}+ds\;,x_{2}-ds-1\right)-S_{t}\left(x_{1}-ds-1,x_{2}+ds\right)\right). \end{array}$$

When compared to the NL-Means approach, the overall complexity of the algorithm has been greatly lowered. At the same time, the offset is considered a cyclic determination condition to reduce space complexity. Rather than computing all of the integral pictures at once, each computation just has to get an integral image of the offset in one direction of the offset. We must, firstly, extend the image before filtering because each filter point in the original image requires a whole search window and many field blocks. The search window is typically half the size of the neighborhood block plus half the size of the expansion.

The INLM filtering algorithm is used in tomato disease images, and its specific steps are as follows:

  Step 1: enter the tomato disease image to be filtered and convert it to a grayscale value
  Step 2: determine the domain window, search box size, and expand the image
  Step 3: take a point in the search block, and take the search block with the point *y* as the center and the search block with the first x in the image as the center to obtain *w*(*x*, *y*)
  Step 4: repeatedly take the next point *y* of the search block and repeat the *c* operation until the point of the search block is traversed
  Step 5: assign the maximum weight to point *x*, normalize the weight, and pass *NL*[*v*(*x*)] to get the pixel value of the first point of the new image
  Step 6: take the second point *x* of the original image and repeat the *c* operation until the entire image is traversed
  Step 7: obtain an image of tomato disease after INLM filtering

As shown in Figure 1, the filtering results reveal that the images filtered using the INLM algorithm retain several features of the original image while lowering the noise. In 3.3.2, the testing results indicate that the INLM algorithm operates ten times faster than the NL-Means algorithm.

### 2.3. DCCAM-MRNet

**Figure 3:**
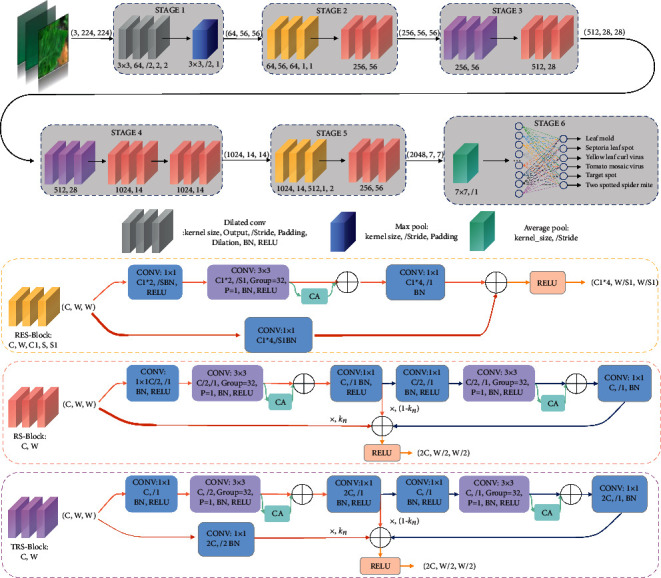
Architecture of the DCCAM-MRNet.

Traditional convolutional neural networks cannot quickly identify diseased spots on tomato leaves because of their small size, lack of feature information, and relatively dispersed feature distribution. Deep neural networks must be utilized to extract more detailed features. As a result, the neural network we choose must have a sufficient number of layers to avoid the problem of gradient disappearance. Simultaneously, it must have the advantages of portability and quick training speed. In 2017, Pant et al. [24] proposed ResNeXt that incorporated the repetition strategy of ResNet and coupled it with the split-transform-merge strategy of the inception family. The residual error mechanism of ResNeXt can solve the problem of gradient disappearance, and when the number of parameters is the same, the recognition effect of ResNeXt is better than that of ResNet. All of the inception modules in the inception family have been meticulously designed [25]. Although the recognition result is satisfactory, several hyperparameters must be manually modified, and portability is lacking. Because of the topological structure of ResNeXt submodules, ResNeXt requires fewer manual modification parameters. After careful consideration, we proposed the DCCAM-MRNet, and Figure 3 depicts its network architecture. DCCAM-MRNet employs ResNeXt50 as its backbone network and replaces the original 7 × 7 convolution kernel with dilated convolution. It has a broader receptive field and improves extracting features without changing the parameters. Between each 3 × 3 and 1 × 1 convolution, a coordinate attention mechanism is introduced. The coordinate channel attention mechanism can evaluate the relationship between channels and position information simultaneously and target the diseased area more precisely, giving it more high weight. Lastly, the DCCAM-MRNet is formed by the RS-Block and TRS-Block of the mixed residual connection method while retaining the RES-Block of the ResNeXt residuals. This combination makes the extraction of features between adjacent layers tighter and achieves higher learning accuracy while compressing the network.

#### 2.3.1. ResNeXt

The main advantage of the ResNeXt is that it does not require deliberate construction of each portion of the network structure details, and it finishes complex classification jobs by the simple stacking of modules, which is relatively concise and easy to transplant. ResNeXt absorbs the advantages of group convolution [26]. The structure of ResNeXt is similar to that of the inception network [27, 28], which links contextual information spatially. It enhances network accuracy without increasing parameter complexity and minimizes the number of hyperparameters employed in the network. However, unlike the inception network, ResNeXt employs the same topology of parallel stacking, which is the same modules as ResNet, to extract features before merging the modules to limit the danger of overfitting.

#### 2.3.2. Dilated Convolution

**Figure 4:**
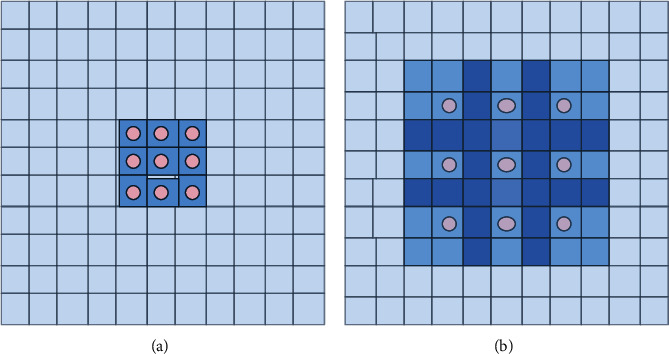
Dilated convolution with different rate. (a) Ordinary convolution (*r* = 1). (b) Dilated convolution (*r* = 2).

The disease features of tomatoes are not obvious, and disease spots are dispersed. As a result, expanding the receptive field is critical. The distinction between dilated and typical convolution is that dilated convolution introduces a new parameter known as expansion rate [29]. The receptive field is enlarged without affecting the size of the feature map by injecting holes into the ordinary convolution. We introduce dilated convolution on STAGE 1 of the DCCAM-MRNet to replace the original 7 × 7 convolution, as shown in Figure 4. Assuming that the size of dilated convolution kernel is *k* × *k* and the expansion rate is *r*, then the actual size of the convolution kernel is as follows:

(6) $$\begin{array}{c} K=r\times \left(k-1\right)+1. \end{array}$$

After dilated convolution, the relationship between the size of the input and output feature maps is as follows:

(7) $$\begin{array}{c} W_{2}=\frac{W_{1}+2p-r\times \left(k-1\right)-1}{s}+1. \end{array}$$

Among them, *W*_1_ and *W*_2_ represent the size of the input and output feature maps, respectively, the step-size is *s*, and *p* represents the patch.

#### 2.3.3. Coordinate Attention

**Figure 5:**
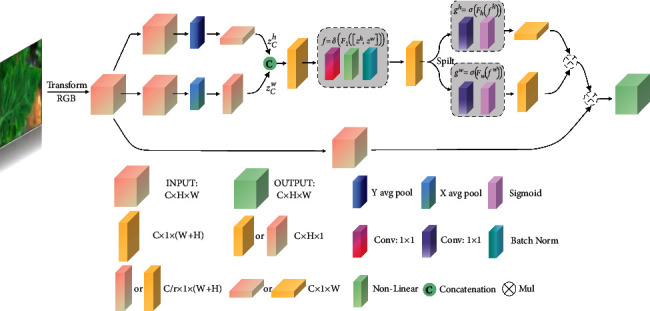
Coordinate attention structure.

Attention mechanisms used in deep neural networks can provide good performance improvements [30, 31]. The SENet model builds a network model from the perspective of the correlation of feature channels, which enhances the directivity of the features extracted by the convolution layer by strengthening the features of essential channels in feature mapping and weakening the features of unimportant channels. Wen et al. [32] embedded SENet into the ResNet-50 network [33], and on this basis, they identified five tomato diseases and achieved 89% detection accuracy. However, the limitation of SE is that only internal channel information is considered, and the importance of location information is ignored. Therefore, CBAM proposed by Woo et al. [34] tried to introduce location information by global pooling on the channel. Still, this method only captures local information and does not pay much attention to location information. To take account of the location relationship based on channel attention, Hou et al. [35] proposed coordinate attention, which is structured as shown in Figure 5. It decomposes channel attention into two feature coding processes, namely vertical and horizontal directions, integrating features with two spatial directions. With this processing, remote correlation can be captured in a spatial direction, while accurate location information can be maintained in another spatial direction.

The specific implementation method is as follows:

For a given input feature *X*=[*x*_1_, *x*_2_,…, *x*_*c*_] ∈ ℝ^*C*×*H*×*W*^, two spatial extents of pooling kernels (*H*, 1) and (1, *W*) are used to code channels along with the horizontal and vertical directions, respectively. The output of Channel *C* at height *H* can be formulated as follows:

(8) $$\begin{array}{c} z_{C}^{\text{h}}\left(h\right)=\frac{1}{W}\sum_{0\leq i<w}x_{C}\left(h,i\right). \end{array}$$

Similarly, the output of channel *C* with a width of *W* can be written as follows:

(9) $$\begin{array}{c} z_{C}^{\text{w}}\left(w\right)=\frac{1}{H}\sum_{0\leq \text{j}<\text{H}}{\text{x}}_{C}\left(j,w\right). \end{array}$$

After generating a pair of direction-aware feature maps, the concatenation connection operation is performed on the spatial dimension, and then the shared 1 × 1 convolution transformation function *F*_1_ is used to get the following:

(10) $$\begin{array}{c} f=\delta \left(F_{1}\left(\left[z^{h},z^{w}\right]\right)\right), \end{array}$$

[·, ·] denotes a concatenation operation along the spatial dimension, *δ* is a nonlinear activation function, and *f* ∈ ℝ^*c*/*r*×(*H*+*W*)^ is an intermediate feature mapping that encodes spatial information in the horizontal and vertical directions. Here, *r* is the reduction rate that controls the block size.

Splitting *f* into two independent tensors *f*^*h*^ ∈ ℝ^*c*/*r*×*H*^ and *f*^*w*^ ∈ ℝ^*c*/*r*×*W*^ along the spatial dimension, two 1 × 1 convolutions are used to transform *f*^*h*^ and *f*^*w*^, respectively, so that they remain tensor with the same number of channels as the input *X*.

(11) $$\begin{array}{c} g^{h}=\sigma \left(F_{h}\left(f^{h}\right)\right),\\ g^{w}=\sigma \left(F_{w}\left(f^{w}\right)\right), \end{array}$$

*σ* is the sigmoid function. *F*_*h*_ and *F*_*w*_ are two 1 × 1 convolutions. *g*^*h*^ and *g*^*w*^ are the weights in two dimensions.

Finally, the weights *g*^*h*^ and *g*^*w*^ in the two dimensions are fused with the input *X* to obtain the output of the coordinate attention block *Y*, which is expressed as follows:

(12) $$\begin{array}{c} y_{C}\left(i,j\right)=x_{C}\left(i,j\right)\times g_{C}^{h}\left(i\right)\times g_{C}^{w}\left(j\right)\text{.} \end{array}$$

#### 2.3.4. Mixed Residual Connection Method

**Figure 6:**
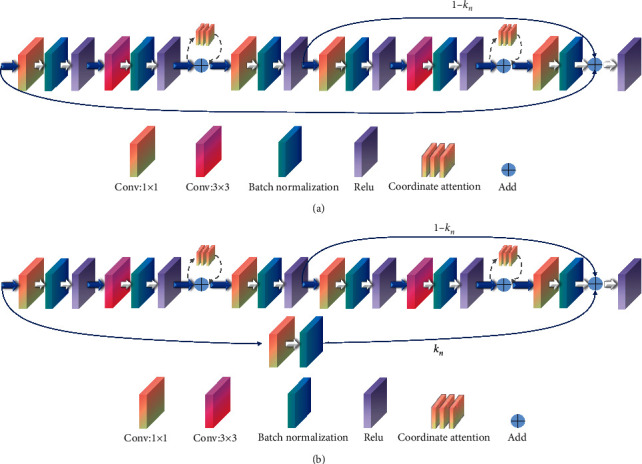
The method of mixed residual connection. (a) RS-Block. (b) TRS-Block.

The trainability of deep neural networks has always been a significant issue. RoyChowdhury et al. [36] were among the first to apply the mature numerical solution of differential dynamical systems to neural network learning. The mixed residual connection method, based on the Adams method of the numerical solution of differential equations, is used for network design in this paper.  Figure 6 depicts two forms of the mixed residual connection method used in this paper: RS-Block and TRS-Block. This method enhances network performance and increases the tightness between adjacent layers for feature extraction by weighted summing the adjacent residual blocks with the current residual block.

In the DCCAM-MRNet, RS-Block is used in the latter part of STAGE 2, STAGE 3, STAGE 4, and STAGE 5. TRS-Block is used at the beginning of STAGE 3 and STAGE 4. The difference between them is that the input channel of TRS-Block is C and the output channel is 2C. Hence, the number of channels is inconsistent and cannot be directly added. Therefore, the input channel needs to be convolved with 1 × 1 to change its channel number to 2C.

Based on Adam's method, the specific steps are as follows:

(13) $$\begin{array}{c} h_{n+m}=h_{n+m-1}+\Delta h\sum_{i=0}^{m}\beta_{i}f_{n+i}. \end{array}$$

In formula (13), Δ*h* is the step-size. *h*_*t*_ ∈ *R*^*D*^ is the output at time *t*. D represents the dimension of the output. *β*_*i*_ is the corresponding weight of *f*_*n*+*i*_, and it satisfies the condition of ∑_*i*=0_^*m*^*β*_*i*_=1. *f*_*n*+*i*_ is the value entered in the layer of *n*+*i*.

In this paper, the mixed residual connection method sets *m*=0 and *β*_*m*_=0 in formula (13). Then, formula (13) becomes as follows:

(14) $$\begin{array}{c} h_{n+2}=h_{n+1}+k_{n}f_{n}+\left(1-k_{n}\right)f_{n+1}. \end{array}$$

In (14), *k*_*n*_ ∈ *R* is the weight coefficient corresponding to the information content in the hidden layer. Especially when *m*=1, it is the Euler method in the differential numerical solution. In this paper, we let *k*_*n*_=0.5, which means that the importance of information in all hidden layers is the same.

## 3. Results

### 3.1. Experimental Environment

The hardware environment of this experiment is Windows (64bit) operating system, Intel Core I7-9700U CPU, and NVIDIA RTX 2080Ti GPU. The programming environment of the INLM filtering algorithm is MATLAB 2020b. The programming environment of the DCCAM-MRNet is Python 3.8.12, Pytorch 1.8.2, and CUDA 10.2. In this experiment, stochastic gradient descent was used to train the DCCAM-MRNet. The batch size of training samples was set to 32, and 8 for test samples. The learning rate Ir was set to 10^−3^, and the *epochs*  was set to 140. The Adam optimizer was used during training, and the cross-entropy loss was used as the loss function.

### 3.2. Effectiveness Experiment of the Module

#### 3.2.1. Effectiveness Experiment of Preprocessing

To test if the preprocessing of tomato leaf disease datasets can increase recognition accuracy, we fed the original dataset and the preprocessed data set, including the dataset expansion and filtering process, into ResNeXt50, ResNeXt50-CA, and DCCAM-MRNet, respectively, to conduct the experiments.  Table 8 displays the recognition accuracy of the original dataset and the preprocessed dataset in three different types of networks. The results reveal that the three networks' recognition accuracy in the preprocessed dataset is greater than that in the original data set. It is because the data set is extended by cropping, flipping, zooming, and brightening, which increases the diversity of the dataset while avoiding the network coverage. The INLM filtering algorithm efficiently reduces the complicated background and removes fuzzy features, resulting in more apparent image features. As a result, following preprocessing, the accuracy of the dataset has increased in all three models.

#### 3.2.2. Effectiveness Experiment of INLM Filtering Algorithm

To demonstrate that the INLM filtering algorithm has a faster convergence speed than the NL-means filtering algorithm, we randomly select 100 disease images from the dataset for filtering in MATLAB and calculate the average time spent by 100 images in the NL-means filtering algorithm and the INLM filtering algorithm. Table 1 shows that the convergence speed of INLM is ten times faster than that of NL-means.

#### 3.2.3. Effectiveness Experiment of Dilated Convolution

In the DCCAM-MRNet, we used dilated convolution at STAGE 1. In the same test environment, we conducted experiments on ResNeXt50, ResNeXt50-Dilated Conv, and DCCAM-MRNet to validate their impact on classification performance. Table 2 demonstrated that utilizing dilated convolution in the ResNeXt50 could improve the accuracy of the network.

#### 3.2.4. The Effectiveness Experiment of Coordinate Attention

To more intuitively understand the improvement in accuracy induced by coordinate attention, we trained and tested the preprocessed dataset using ResNeXt50, ResNeXt50-SE, ResNeXt50-CMBA, ResNeXt50-CA, and DCCAM-MRNet. Table 3 displays the accuracy of different networks on the test set. The experimental results showed that the three networks using the attention mechanism improved 0.9%, 2.3%, and 3.6%, respectively, in terms of accuracy compared to ResNeXt. The CA attention mechanism outperformed the other attention mechanisms in terms of improving accuracy. The accuracy of the DCCAM-MRNet proposed in this paper is 94.3%, which indicates that the tomato leaf disease features are deeply extracted, and the network is effective in identifying.

#### 3.2.5. Effectiveness Experiment of Mixed Residual Connection Method

We measured the number of parameters of ResNeXt50, ResNeXt50-MRC, ResNeXt50-LM, ResNeXt50-CA, and DCCAM-MRNet in terms of model compression. The results are displayed in Table 4, indicating that the number of parameters in the ResNeXt50-MRC is 0.35 M less than that 22.68 M of ResNeXt50-LM. The experiment result shows that MRC is superior to LM in model compression. The number of parameters in the DCCAM-MRNet is 0.11 M less than that of ResNeXt50.

### 3.3. Ablation Experiment

To thoroughly validate the effectiveness of the method proposed in this paper, we employed the same dataset and experimental environment in each experiment, only changing the components that needed to be compared. The backbone network in the ablation experiment is ResNeXt50, and the performance of several schemes is compared by adding one or more of the three methods of DC, CA, and MRC.  Table 5 displays the comparing results.

Table 10 shows that the DCCAM-MRNet has higher accuracy than other networks, reaching 94.3 percent. When the coordinate attention is given to ResNeXt50, it enhances its accuracy by 3.6 percent when compared to the initial ResNeXt50. Similarly, the ResNeXt50 with Dilated Conv or Mixed Residual Connection outperforms the original ResNeXt50 by 1.7 percent or 0.9 percent, respectively. According to the evidence shown above, all three strategies are successful at increasing accuracy.

The number of parameters in the Dilated Conv network is the same as in the single variable network, which is consistent with the premise that Dilated Conv does not change the number of parameters. ResNeXt50 with mixed residual connection technique has 0.67 M fewer parameters than ResNeXt50 without mixed residual connection method, suggesting that the mixed residual connection approach aids in network compression.

### 3.4. Overall Evaluation of the DCCAM-MRNet

**Figure 7:**
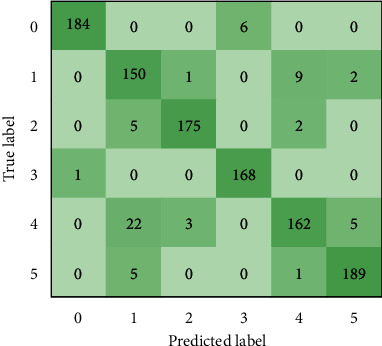
Confusion matrix of the DCCAM-MRNet.

In the same test scenario, the DCCAM-MRNet outperforms its backbone network ResNeXt50 in terms of learning stability in the learning process and recognition accuracy.  Figure 7 depicts the performance of DCCAM-MRNet in each category. The numbers 0, 1, 2, 3, 4, and 5 in the confusion matrix represent the six diseases, namely the leaf mold, Septoria leaf spot, yellow leaf curl virus, tomato mosaic virus, target spot, and two-spotted spider mite, respectively. The test set contains 1092 images in total, however, only 1090 of them are used in this test and displayed in the confusion matrix. The number of accurately predicted images, 1028 in total, is shown in the diagonal of the confusion matrix. The overall recognition accuracy of DCCAM-MRNet is 94.3%. Table 10 displays the accuracy of disease recognition in the DCCAM-MRNet for six different diseases. It can be seen that the highest recognition accuracy for the network is the tomato mosaic virus, reaching 99%, while only 84% for the target spot.

### 3.5. Comparison with Other Networks

We employ four indexes to evaluate the performance of the network: recall, F1-score, precision, and mAP. Table 6 displays the results. The indexes of DCCAM-MRNet surpass 90%, which is higher than those of other networks, showing that this network has more advantages and a more robust recognition effect for tomato leaf diseases than other networks.

### 3.6. Performance on the Plant Village Public Dataset

**Figure 8:**
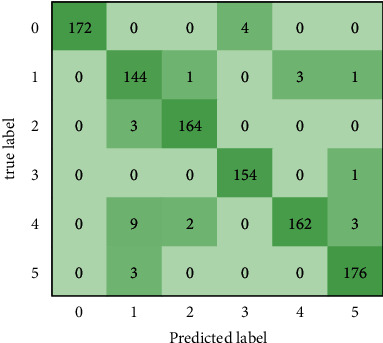
Confusion matrix of the DCCAM-MRNet.

As experimental data, 1000 images of tomato leaf diseases from the plant village public dataset [37] are used. For recognition, the disease dataset is sent into the DCCAM-MRNet. Consequently, the recognition accuracy on the plant village public dataset is 97.0%, while the recall and F1-score are 97.6% and 97.1%, respectively.  Figure 8 depicts the confusion matrix. The recognition accuracy for tomato mosaic virus using the public dataset is 92%, which is 8% higher than that obtained using our dataset. The recall and F1-score are also higher than those obtained using our dataset.

## 4. Discussion

To identify tomato leaf diseases, we build a DCCAM-MRNet in this article. In experiment 3.7, we use the DCCAM-MRNet to identify the public dataset plant village, and the accuracy is 97.0 percent. When we use our dataset, we get an 8% better recognition accuracy for the tomato mosaic virus. The tests indicate that integrating the INLM and DCCAM-MRNet for tomato leaf disease identification is effective and capable of tackling the problem of low accuracy in tomato leaf disease identification to some extent, while more research is needed. (1) In this research, the DCCAM-MRNet is connected using the mixed residual connection method, with the weight coefficient of information amount in the hidden layer equal to 0.5. However, because the importance of information quantity varies in different hidden layers, it should be a floating number, and the mixed residual connection method should be adjusted to accommodate for floating, resulting in a superior network compression impact. (2) Current research focuses on identifying a single disease type on a single leaf, with less emphasis on identifying many illnesses on the same leaf, which has limits. Extracting traits and identifying mixed illnesses on tomato leaves will require more research. (3) This paper's data on leaf disease is insufficient. To improve the model's generalization capacity, the image data of tomato leaf diseases should be gradually added in the future.

## 5. Conclusions

For the complex tomato planting background, inconspicuous tomato leaf disease features, and distributed disease spots, a method for tomato leaf disease identification based on the INLM and the DCCAM-MRNet model is proposed. Firstly, a tomato leaf disease classification dataset with 10,923 tomato leaf images is generated. Secondly, the INLM filtering algorithm filters the tomato leaf disease dataset to reduce the influence of complex tomato planting background and blurred disease features on the images and improve image quality. The INLM filtering method is ten times faster than the traditional NL-Means filtering algorithm in terms of filtering speed. Then, for tomato leaf diseases with obscure disease features and scattered spots, a novel neural network DCCAM-MRNet is developed using the ResNeXt50 as the backbone network. Dilated convolution and coordinate attention methods are used in the DCCAM-MRNet to improve the extraction of subtle and scattered feature points. The mixed residual connection method is used to enhance the tightness between adjacent layers for feature extraction, which reduces the number of network parameters and improves the learning accuracy of the network. The final experimental results demonstrate that the DCCAM-MRNet has an accuracy of 94.3% in identifying tomato leaf diseases. In addition, the number of parameters decreased by 0.11 M compared to the ResNeXt50 backbone network, which aids in network compression.

Tomato and other crop leaf disease recognition is still a hot research area in image recognition technology. The DCCAM-MRNet may be used for disease recognition after capturing tomato leaf pictures, which is critical for preventing and controlling tomato leaf diseases and ensuring tomato productivity and quality. The next step in this paper's research will be to see how the network can handle more types of tomato leaf illnesses and how to increase the network's disease recognition accuracy by enhancing the extraction of small, scattered data. In addition, we must investigate how to reduce the network's size to improve the detection of tomato leaf diseases and ensure agricultural productivity.

## Figures and Tables

**Table 1:** Time spent by NL-Means and INLM filtering method.

Denoising algorithm	NL-means (s)	INLM (s)
Average time	45.63	4.32

**Table 2:** The accuracy of three networks.

Network model	Parameters (M)	Accuracy (%)
ResNeXt50	23.00	86.6
ResNeXt50-DC	23.00	88.3
DCCAM-MRNet	22.89	94.3

**Table 3:** The influence of coordinate attention on network accuracy.

Network model	Accuracy (%)
ResNeXt50	86.6
ResNeXt50-SE	87.5
ResNeXt50-CMBA	88.9
ResNeXt50-CA	90.2
DCCAM-MRNet	94.3

**Table 4:** Comparison of model parameters.

Network model	ResNeXt50 (M)	ResNeXt50-MRC (M)	ResNeXt50-LM (M)	ResNeXt50-CA (M)	DCCAM-MRNet (M)
Parameters	23.00	22.33	22.68	23.94	22.89

**Table 5:** Comparison of recognition accuracy and parameters of different networks.

Network model	Parameters (M)	Accuracy (%)
ResNeXt50	23.00	85.6
ResNeXt50-DC	23.00	88.3
ResNeXt50-CA	23.94	90.2
ResNeXt50-MRC	22.33	87.5
ResNeXt50-DC-CA	23.94	93.1
ResNeXt50-DC-MRC	22.33	89.6
ResNeXt50-CA-MRC	23.17	92.5
DCCAM-MRNet	22.89	94.3

**Table 6:** Evaluation indexes of the networks.

Network model	Recall (%)	F1-score (%)	Precision (%)	mAP (%)
MobileNet	78	74	77	71
ResNet50	83	81	80	74
ResNeXt50	87	85	83	77
LM-ResNet	86	86	87	82
InceptionResNetV2	84	80	85	80
EM-ERNet	82	83	85	81
B-ARNet	84	82	86	81
SENet	85	84	86	82
CMBA-ResNet	86	85	88	84
DCCAM-MRNet	94	93	94	90

**Table 7:** Symptoms and image sources of 6 tomato diseases.

Disease type	Disease picture	Early symptoms of the disease	Advanced symptoms of the disease	Data sources
Leaf mold	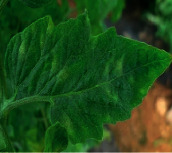	Irregular or elliptical yellowish spots appear on the leaf blade, with indistinct margins of the spots.	The disease spot breeds gray or black irregular-shaped mold layer.	Tomato greenhouse
Septoria leaf spot	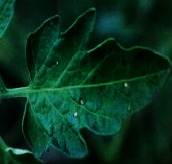	Round or nearly round spots appear on the front and back of the leaf with dark brown margins and many small ink-colored grain spots scattered.	The leaves are covered with spots, and the leaves turn yellow, causing early abscission.	Internet
Yellow leaf curl virus	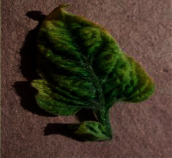	The upper leaves are slightly yellowed and irregularly spotted. Purple veins frequently appear on the abaxial leaf.	The upper leaves and new shoots show symptoms, with small and red opaque	Internet
Tomato mosaic virus	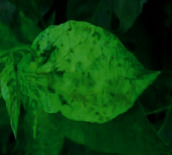	Unevenly mottled shades of green, the leaves do not become smaller, and they do not produce deformities.	Leaf-blade shows yellow-green, flowering leaves are uneven.	Tomato greenhouse
Target spot	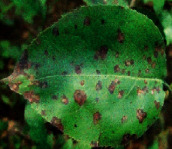	Subround, irregular brown spots on the leaf blade.	The color of the spot deepens, the area of the spot becomes larger, and it leads to leaf perforation.	Tomato greenhouse
Two-spotted spider mite	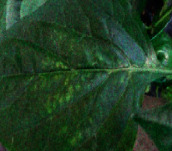	Many tiny greenish spots are scattered in the leaves' middle and lower parts.	The leaves fade to grayish-yellow and fall off.	Internet

**Table 8:** The recognition accuracy of the original dataset and the preprocessed tomato leaf disease dataset in the three models.

Network model	Original data set (%)	Preprocessed data set (%)
ResNeXt50	78.4	85.6
ResNeXt50-CA	84.7	90.2
DCCAM-MRNet	89.1	94.3

**Table 9:** Details of six tomato diseases.

Size of the data set				Division of the data set		
Disease type	Original number	Expanded number	Percentage	Training set (70%)	Validation set (20%)	Test set (10%)
Leaf mold	465	1858	17.00	1300	372	186
Septoria leaf spot	436	1745	15.98	1222	349	174
Yellow leaf curl virus	490	1961	17.95	1373	392	196
Tomato mosaic virus	448	1790	16.40	1253	358	179
Target spot	457	1827	16.73	1279	365	183
Two-spotted spider mite	435	1741	15.94	1219	348	174

**Table 10:** Performance evaluation of each disease.

Disease type	Recall (%)	F1-score (%)	Precision (%)
Leaf mold	99	98	97
Septoria leaf spot	97	98	93
Yellow leaf curl virus	82	87	96
Tomato mosaic virus	93	89	99
Target spot	96	97	84
Two-spotted spider mite	98	97	97

## Data Availability

The data used to support the findings of this study are obtained from [37].
